# Interculturalism: Not a new policy paradigm

**DOI:** 10.1186/s40878-018-0091-5

**Published:** 2018-06-19

**Authors:** Tariq Modood

**Affiliations:** Centre for the Study of Ethnicity and Citizenship, SPAIS, University of Bristol, Bristol, BS8 1TU UK

**Keywords:** Multiculturalism, Interculturalism, Local, National, Micro, Macro

## Abstract

The central question of the symposium has been whether interculturalism provides a new paradigm that transcends multiculturalism? I note that, consistent with my own position, none of the commentaries answers this question in the affirmative. I concur with the view that interculturalist approaches suffer from an indeterminacy in the use of concepts such as local, place and proximity. When such concepts are given specification, they can have two different meanings: a) face to face encounters, b) urban life and/or governance. Whilst (a) and (b) can be connected together, a dichotimising logic is employed by interculturalists relation to the micro-macro and the city-national. I conclude by specifying, by reference to my work, the key features of multiculturalism that a replacement paradigm has to engage with.

## Interculturalism: Not a new policy paradigm

Levrau and Loobuyck (2018) identify the central question of this Special Issue as ‘whether or not interculturalism provides a new paradigm that transcends multiculturalism’ (p. 10). There seems to be general assent that multiculturalism (herafter MC) is focused on what I referred to as the macro and interculturalism (hereafter IC) on the micro (Modood, 2017, pp. 6-7); though different contributors to this symposium use different vocabularies. For example, while MC is based on the idea of intercultural dialogue at the level of public discourse, debates and ideas, IC provides a micro-level focus on interaction largely missing from the former. Yet as Levey (2016) has pointed out, European IC – the IC of this special issue and as represented by authors such as Cantle and Zapata-Barrero – has in the last decade or so come to share the ambition of its Quebecan counterpart – and offer a macro position too.

Hence the question of the symposium indeed is not just whether IC adds to or corrects MC in certain limited ways but whether it is a new paradigm, a new, distinctive and convincing macro theory of its own. Whilst this is Zapata-Barrero’s (2017a) claim and denied by me (Modood, 2017) I do not see any of the commentators giving it an affirmative answer. Joppke (2018) finds IC shallow and dangerous and de Waal (2018) finds it ambivalent and dangerous, while Levrau and Loobuyck (2018), Boucher and Maclure (2018), Oosterlynck (2018), Levrau (2018) and Kastoryano (2018) identify how IC can add to and complement MC without replacing it (and Kastoryano brings transnationalism into the mix).

While Zapata-Barrero is not totally dismissive of MC and thinks that its core emphasis on rights and equality needs to be preserved in some way, he says he wants to jettison what he calls excessive recognition and separate funding streams and make cities and not the state the locus of policy. He is also clear that its not a matter of having some IC policies complement MC policies, rather what is worth preserving about MC will be done within an IC paradigm (also in Zapata-Barrero 2017b). What this preservation will consist of is not clear as he spends more time criticising MC than showing what he is preserving of it. Without belabouring the point I will just remind the reader that one of the charges against interculturalists that I made in my original symposium contribution was that they do not engage with MC theory, advocacy or policies but rather speak about how multiculturalism is perceived. This practice is continued in Zapata-Barrero’s (2017a) contribution to this symposium. Various negative perceptions of multiculturalism are mentioned as part of a case for why a new paradigm has justifiably emerged without saying whose perceptions are being referred to and to what extent the perceptions are correct. For example, he states that those who oppose multiculturalism 'see it as having been imposed by racial and ethnic minorities’ (p. 6) without saying whether he agrees or disagrees with this perception. Sometimes he makes related points in his own voice, as in his aim to ‘regulate the excessive recognition to certain cultures, and limit illiberal practices contravening human rights’ (p. 6) of ‘boundless multiculturalism’ (p. 5).

While all the commentators agree that a focus on the micro/local encounters/policies is an essential feature of interculturalism, they also think its claims amount to more than that. A helpful analysis is provided by Boucher and Maclure (2018, p. 3) who state that Zapata-Barrero’s ‘three pillars of interculturalism are the promotion of contacts between individuals with different cultural attachment, the rejection of a state-centric policy paradigm and the corresponding promotion of the role of cities and the promotion of mainstreaming as opposed to group-targeted measures’.

The first two items certainly seem to fit with Zapata-Barrero’s emphasis on proxmity and his statement that interculturalism ‘primarily promotes face-to-face relations and develops most of its policies at the micro-level’ (pp. 13-14). I suggest, however, that this position has some inter-related problems which have been identified by some of the symposiasts.

The problems I am referring to can be unpacked as follows:

i) What does local/place/proximity mean? There is a fundamental indeterminacy.
ii) When micro and local are given some content, they seem to have at least two very different meanings – a) face to face, b) city;
iii) And whilst (a) and (b) can be connected together, a completely different logic is apparent in relation to the micro-macro and the city-national, namely a dichotimising logic.

## What does ‘proximity’ or ‘micro’ mean?

What does local/place/proximity mean? As Joppke (2018) rather caustically but devastatingly notes, strictly speaking it is vacuous (p. 7). We can rehearse his arguments by commenting on Oosterlynck (2018) because he is open to a similar critique (even though his piece makes some very good points, see below). Explaining an analytical shift in focus away from nation-states, he says the purpose is to emphasise ‘*places*, in a relational rather than territorial sense’ (p. 3; itals in the original). But this by itself does get us to the micro as France is a place, not just Paris or Montmartre – and this is consistent with thinking of a place in a ‘relational’ sense. So why does he, like Zapata-Barrero and ICs, think ‘place’ is about something small scale, about something smaller than a country? Moreover, a nation-state is not just a territory in the way that a mountain range is, it is a set of highly complex institutions and relations over time as well as over territory. The additional suggestion that ‘proximity’ is ‘*the* defining feature of place as a principle of socio-spatial structuration’ (Oosterlynck, 2018, p. 3, itals in the original; cf. Zapata-Barrero, 2017a, b, pp. 13-14) is not much more helpful, as that too is not a feature of place per se (eg., it is not a feature of a large place) but of small scale. Though it leaves unclear how small: street? neighbourhood? city? This simply takes us back a la Joppke to the problem of indeterminacy in relation to the use of spatial terms without signifying scale. Imagine if someone said ‘integration is about time’ without specifying whether we are talking about months, years or decades.

Relatedly, Oosterlynck (2018) says he wants to get beyond a priori privileging and normativity. He rightly identifies my work as normative but thinks his own is not. That’s not how it seems to me. In his own work his use of ‘solidarities’, ‘common projects’ and ‘positive feelings’ (p. 4) ‘humanitarian’, ‘misrecognised’, ‘struggle’ (p. 5) and ‘shared responsibility’ (p. 8) are all normative; in the first instance they are about normativities of his subjects or places but in so far as he promotes some and not others, they are about his own normative position. While he wants ‘to move beyond the a priori privileging of certain sources of solidarity’ (p. 5) and I can see the value of that, I want to caution against (as I take Joppke (2018) to be doing so too) an a priori privileging of certain places by calling them ‘places’ and excluding other places such as countries or states. To say we should focus on ‘the concrete places where [people] live, work, learn and play together in superdiversity’ (p.8) seems to be a form of clear privileging; it is not a conclusion of an empirical inquiry but an argument about what should be studied and so is a priori. There may be some value in this methodological localism but surely it – as a set of concepts challenging another set of concepts - is no less of an a priori privileging than any other methodological ‘ism’.

Once terms such as ‘place’ are given some preferred content, we can see how it can be used to adjudicate the argument between MC and IC over the latter’s claim that it but not MC is dialogical. Boucher and Maclure (2018) rightly remind us that ‘[MC] dialogue.. refers to the meeting of ideas, values, symbols and arguments, whilst intercultural contact refers to the meeting of real people in specific physical places’. And they usefully add ‘[o]f course the two notions are not logically mutually exclusive, but they do not imply one another… Most importantly, emphasising the value of one or the other may command different policies. The importance of intercultural dialogue is likely to require that the government is more sensitive to the values and practices of minority groups expressed by their elites in various formal settings (courts, official public consultations, legislative assemblies and so on), whereas the importance of intercultural contacts is likely to require policies of urban planning emphasizing the creation of urban spaces conductive to positive intercultural encounters’ (p. 4).

Whilst many exponents of ‘intercultural contacts’ (aka ‘everyday multiculturalism’) are content with ‘rubbing along’, ‘indifference’ and ‘civility’ (usually without acknowledging that such cordial and peaceful social relations rely on an appropriate state structure and civil society so that these things can happen and produce positive rather than negative encounters and relationships.) Levrau (2018) highlights the importance of developing individual citizen virtues to be practiced in such relations because ‘the justice of a society not only depends on the basic structure, but also on the many (daily) actions and choices of citizens’ (p. 2). He thinks that Zapata-Barrero and IC bring the importance of the interaction between ordinary citizens to the fore and so are better placed than MC to develop the necessary social ethos (p. 8). Perhaps he is right to some extent but that’s not what Zapata-Barrero’s IC – with its focus on post-MC governance of cities, diversity as a competitive advantage and reversing excessive recognition of minorities – seems to be about. Indeed, Levrau’s explicit use of the Rawlsian phrase, ‘the basic structure’ is a reference to a state-based system of justice, with no special connexion to city-level governance. In any case I presume Levrau would agree with de Waal (2018) that a MC state and IC citizens are not an either-or but complementary (p. 2). This leads to the second problem with Zapata-Barrrero’s framework, one which is implicit in the second ‘pillar’, namely, the rejection of a state-centric policy paradigm and the corresponding promotion of the role of cities.

## The Dichotimising logic of Interculturalism

The problem I identify here is the dichotimising logic of IC in the use of the micro-macro and city-national as either-or binaries. The commentaries show the limitations of this binary logic in two ways, in relation to group identities, and in relation to the effectiveness of diversity policies.

The interculturalist argument that identities and solidarities are lived in a local context, in neighbourhoods, everyday encounters and so on are illustrated by some valuable examples from Belgium offered by Oosterlynck (2018). The value of the examples is that they nicely challenge the false dichotomy of local-national. Oosterlynck widens our framework for understanding concepts such as group solidarity by showing how different considerations come into play in different contexts (eg. efficiency, culture, humanitarian) and especially that it makes no sense to privilege the micro over the macro or vice versa. I am pleased to have his support for my insistence that ‘a micro-level focus should not lead us to neglect macro-level processes and the reality of groupings’ (p. 6). He is clear that in empirical inquiry a ‘micro-level focus does not mean that one ignores the macro-level processes since the latter very much structure the places of everyday life in diversity’ (p. 8). Judicious remarks are offered on how to include both macro- and micro-level processes and his illustrations in relation to these and the large set of Belgian projects that they are from are most apt in researching both levels in a complementary way. I particularly welcome Oosterlynck’s affirmation that it is not only macro-level understanding that is able to and must perceive group identities or groupings but that the same conceptual resources are necessary for micro-level processes (eg in relation to workplaces such as Tower Automotive, p.5).

Relevant here is a recent study by Antonsich (2018) of non-white migrants and second generation individuals in Italy reference the local and the national in talking about their hybridic identities and in resisting ‘othering’. Through a number of cases he bring out how ‘[f]irst, participants blurred the distinction between these two scales, as identitification and attachment to local places were narrated by also mobilizing national markers. Second, the sense of local rootedness of the participants was not cast against the nation, but it was strategically deployed to claim a place in the nation.’ (p.1).

This might actually be the appropriate moment to bring in another possible duality, namely that between a territorial or at least a country-based nationalism, including multicultural nationalism, and a transnational nationalism as deployed by Kastoryano (2018). She argues that the third generation based on migration origins, say Turks in Western Europe, are not identifying with the states in which they are citizens but with a pan-Turkish nationalism, deliberately promoted by the Turkish state but also arising from how such individuals perceive themselves as located in the global order. Certainly there is a phenomenon of transnational and diasporic identities, including those, such as membership of the Muslim ummah or the black Atlantic, which are not state-based and state-promoted. It may even be the case that some of those identities are rising in importance, or at least not fading away, but they certainly do not have to be in an exclusivist binary relation with national identities such as say German or French, an observation which I hope can be accepted as commonplace now. In fact, it is even possible that such transnational identities may be explicitly called upon to support multicultural nationalistic integration. This was the finding of Dikici (2016), who studied how The Dialogue Society, a strand of the transnational Gulen movement, played an active role in the cities of London and Bristol and Britain more generally in promoting hyphenated British-Turkish identities within a British multiculturalism.

The dependence of the local on the national is not just in terms of group identities. As Joppke (2018, pp. 7-8) points out it is politically dangerous to vacate the national. It is not only the case that most policies in relation to cultural diversity and integration take place at a national level, but national level laws and policies usually trump those at a local level when they are not in alignment (though there is sometimes scope of differential implementation across a national territory). As he points out, nearly all burka bans are national not local (though interestingly the 30 burkini bans in the south of France were enacted at a local level – and overturned by national laws). Not only can the burqa bans only be overturned at a national level only but the burkini bans too could only be reversed by national laws.

Boucher and Maclure (2018) note that the original exponent of ‘the contact hypothesis’, Gordon Allport, realised not any kind of contact is good. ‘In addition to the equality of status condition, Allport adds that, to generate positive outcomes, contact must benefit from institutional support, must be sustained and must happen between people who have common goals’ (p. 6). It is true that municipalities have a role to play here but ‘most of the triggering conditions above-mentioned are items that the state usually provides and secures.’ (p. 6). I am in full agreement with Boucher and Maclure’s conclusion that Zapata-Barrero (2017a) vastly underestimates the role of the state in creating the conditions for intercultural contact and its success. Indeed, I do not see any of the commentators disagreeing here. This does not by itself mean that the state level policies should be multiculturalist (or indeed IC). Simply that to say that IC is about policies at a city level is not to have offered a substitute for multiculturalism, let alone a new paradigm but at best to offer a difference in emphasis.

## New lamps for old?

As for the third pillar of IC in the Boucher and Maclure (2018) list above, namely, the promotion of mainstreaming as opposed to group-targeted measures. Their view is sound. We cannot give up on group-specific policies in favour of mainstreaming because ‘group-specific policies may be necessary to correct patterns of discrimination and injustices’ (p. 7). Having already highlighted arguments for differential needs in my first contribution to the symposium, I do not think there is anything I need to add here**.**

There is actually very little in any of the six commentators’ remarks on interculturalism that I disagree with (even my quibbles with Oosterlynck about IC’s relation to the a priori are not really about IC as such, especially as I have said above I find his observation in relation to the Belgian cases insightful and helpful to my case). I do not see that these comments amount to a replacement paradigm. I want therefore to underline my opening point: none of the commentators offer any support to Zapata-Barrero’s (2017a) claim that IC is a replacement paradigm. I will conclude therefore by re-visiting the question of if IC was to be a replacement of MC, what would it be replacing?

My answer to this question can perhaps be gleaned from my initial contribution to this symposium (Modood, 2017). Let me directly yet briefly outline what I see myself as having argued under the term ‘multiculturalism’ over nearly three decades, so that readers may judge to what extent Zapata-Barrero’s new ‘paradigm of interculturalism’ is a replacement of and to what extent it is complementary with it; to what extent it is additive or subtractive. This can also serve as my contribution to Levrau and Loobuyck’s (2018) outlining of the various positions in the introduction to the commentaries. They distinguish between on the one hand a Quebecan majoritarian IC, especially the position of Bouchard, and a more cities-based IC of Zapata-Barrero and the European institutions such as the Council of Europe and the European Commission; and on the other hand between a liberal nationalism of Kymlicka and a more multicultural nationalism of Parekh (Levrau & Loobuyck, 2018, pp. 3-10).

My work clearly falls in the latter category and so perhaps the key features of the multiculturalism that I developed or contributed to, and so which might at least in part be taken as the old paradigm that is supposed to need replacing are:

1. I came to racial equality policy work at a local government level (London Borough of Hillingdon) in 1986–87 (and to research in 1991, Nuffield College, Oxford) and against the British anti-racist orthodoxy of the time I insisted that oppressed or marginal groups should be able to define themselves and that this was an essential feature of racial equality. Initially I thought in terms of ethnic pride as essential to uplift, deriving this idea from my understanding of currents within the African-American struggle for group pride and solidarity from the mid/late 1960s, partly associated with political radicalism but also with wider currents as expressed in popular slogans such as ‘black is beautiful’. Shortly afterwards, I saw this emphasis on identity in terms of a search for equality that goes beyond individual rights and sameness of treatment to a respect for difference, as in Charles Taylor’s concept of ‘recognition’ and Bhikhu Parekh’s multiculturalist communitarianism. I have tried to apply this perspective to the following areas:
2. Collective struggle and self-emancipation (ethnic assertiveness): eg., in resisting the subsumption of British Asian identities into a political ‘black’ identity without the consent of Asians; eg., insisting that Muslims be accepted qua Muslims as a minority if that is the identity they wished to prioritise as a public identity in Britain and opposing those who argued that religious identities had no place in anti-racism or in British politics more generally. In this way I saw that the transition from racist ascription to ethnic identities had to be be extended to religion as a dimension of racialised ethno-religious identities and the struggle for recognition. More generally, I saw group pride mobilisation as a form of integration through identity assertiveness and political movements at macro and symbolic levels and so paving the way for egalitarian integration at other levels.
3. Liberal anti-discrimination, anti-incitement to hate laws and policies: I urged these be made sensitive to cultural racism, Islamophobia, religious discrimination/harassment and the ethnic/religious ‘penalty’.
4. Social mobility: I researched ethnicity not just as an obstacle or a ground of discrimination but also as an advantage; focusing particularly on entry to higher education and came to develop the concept of ethnic capital to explain why British Asians were becoming over-represented in British universities.
5. Minority religions should not be understood in terms of doctrines only but also as ethno-religious groups and can be accommodated in a principled yet pragmatic way by drawing on Western Europe’s moderate political secularism as state-religion connexions, rather than naïve ideas of separation or neutrality.
6. National Identity: From early on I viewed national identity as essential to full egalitarian belonging but if it was to be inclusive of minority identities it could not simply be a civic or political/institutional identity, yet nor be simply based on a top-down majoritiarian/dominant historical narrative or list of values but had to be woven in multilogues and inevitably sometimes through controversy, such as for example the Rushdie affair (for all six points see Modood 2007/2013; and for their relation to a wider Bristol School of Multiculturalism, see Levey, 2018, in press).

I believe I have made it abundantly clear in this symposium as well as in previous writings that multiculturalism has something to learn from interculturalism, and that this mainly focuses on IC giving MC a new micro focus to complement MC’s macro focus (Antonsich, 2016). This is the genuine additive part of interculturalism and of related research currents such as ‘everyday multiculturalism’ and ‘superdiversity’ (Sealy, 2018) – as long as it is not pursued through a dichotomising logic and that does not devalue a macro-national empirical and normative approach. Empirical and normative inquiry at an everyday or local or single institution (eg., a workplace) or city levels in relation to the six items listed above are surely to be welcomed.

On the other hand, I do not think that macro-multiculturalism has been replaced, should be replaced or is about to be replaced by an alternative pro-diversity paradigm. Majoritarian nationalism seems to the dominant politics in so many parts of the world today (in Russia, China, India, many Muslim-majority countries as well as the USA and across Europe) and that classical liberalism, aka culture-neutral civic integrationist individualism is one of the casualties. In this context, multicultural nationalism (Modood, 2017) unites the concerns of some of those currently sympathetic to majoritarian nationalism and those who are pro-diversity and minority accommodationist in the way that liberal nationalism (with its emphasis on individualism and majoritarianism) or cosmopolitanism (with its disavowal of national belonging and championing of open borders) does not. It therefore represents the political idea and tendency most likely to offer a feasible alternative rallying point to monocultural nationalism.

I am grateful to all the symposiasts for their contributions. I have learnt something from each and so the symposium has extended the examination of the relative merits of a version of multiculturalism and a version of interculturalism, in particular the validity of the interculturalist critique of multiculturalism and the consequent claim that interculturalism is a new successor paradigm to multiculturalism. My reading of the symposium is that this latter claim is not substantiated.
